# Height estimation based on second cervical vertebra measured using three-dimensional computed tomographic scanning in Iranian adults

**DOI:** 10.1186/s13104-019-4634-0

**Published:** 2019-09-18

**Authors:** Seyed Reza Saadat Mostafavi, Azadeh Memarian, Omid Motamedi, Mohammadreza Khaleghi, Mehdi Pouromidi

**Affiliations:** 1 Department of Radiology, Iran University of Medical Sciences, Tehran, Iran; 2 Department of Forensic Medicine, Iran University of Medical Sciences, Tehran, Iran; 3 Iran University of Medical Sciences, Tehran, Iran

**Keywords:** Body height, Cervical vertebrae, Tomography, X-ray computed, Linear dimensions

## Abstract

**Objectives:**

The cervical vertebrae are more durable than other skeletal components, and therefore may be the only remnants of a dead body. The present study aims to investigate the role of several linear dimensions of the second cervical vertebrae measured by Three-Dimensional Computed Tomographic Scanning (3D CT Scan) in height estimation of Iranian adult population. In this cross-sectional study, height determination was performed by measuring 15 indexes of the second cervical vertebrae. Indexes were obtained by screening cervical CT scan of 66 patients (33 males and 33 females) aged ≥ 18 years at Rasoul Hospital. Chi square, T student and logistic regression tests were used for statistical analysis. The significance level was considered to be < 0.05.

**Result:**

In the total population, among the indexes for the second cervical vertebrae, the Max height of the axis (AMA) (r = 0.470, P = 0.0001), Max length of the axis (CMA) (r = 0.320, P = 0.007), and Sagittal max body diameter (DSMC) (r = 0.281, P = 0.019) had a strong and positive correlation with height. The results of this study showed the accuracy of linear dimensions of cervical vertebrae in determining the body height of the Iranian adult population.

## Introduction

Forensic anthropology tries to define the identity of corpses. Since bones remain several years after death, the skeletal examination could be especially useful to identify those cases [1, 2]. Height is the main component in the construction of the biological profile of an individual and is one of the basic actions to identify is to determine the height of the individual once remains are found [3, 4]. There are various approaches to use a metrical study of skeletal remains to define the height. However, the best outcomes are obtained from the remains of long bones [3, 5–8]. Though in most of the times, we do not have long bones or they are fragmented, while smaller bones, such as vertebrae, preserve better [9]. Therefore, studies performed on the spinal column to estimate height from the spinal column segments [10, 11].

The relationship between heights of people with spinal vertebrae morphology is one of the effective factors in identifying a person in forensic medicine. For this reason, in cases where only part of the body spine is available, the morphology of the vertebral column can explain the various skeletal features of a person, including his height. Different calculations on the vertebral column in different areas of the neck, chest and lumbar determine various skeletal characteristics of individuals. Each component of the spinal cord at any portion, especially in the cervical area, can be of great help in identifying height, especially in cases where it is not possible to assess height, such as severe traumas [3, 12–14].

However, identification using the skeletal remains is subject to many limitations. For example, in many cases, skeletal remains are not coherent, fragmented or have changed the process of corruption. Also, due to differences in the biology of different individuals, the genetic, ethnic and the environment it is not possible to generalize the result of the studies limited to specific populations to other people [12, 15]. According to our search, there is a limited study on using the indexes of the second cervical vertebrae and there is no study in Iranian population. Therefore, the study aims to estimate the height based on the measurement of the indexes of the second cervical vertebra using Three-Dimensional Computed Tomographic Scanning (3D CT) scan in the Iranian adult population.

## Main text

### Methods

This is a cross-sectional study. In this study 66 individuals (33 male and 33 female) aged ≥ 18 years who underwent cervical vertebrae CT scan in Rasoul Akram Hospital were selected randomly. A randomized sampling method was used and all participants signed informed consent.

The sample size was determined according to Vasavada study in 2008, the mean height of seven cervical vertebrae in male and female were 24.6 ± 2.7 mm and 22.6 ± 2.4 mm, respectively. By considering a confidence interval of 0.05 and the study power of 90%, the required sample size for this study was 33 for male and 33 for female:

$${\text{n}}_{ 1} {\text{ = n}}_{ 2} { = }\left( {{\text{S}}_{ 1}^{ 2} \pm {\text{ S}}_{ 2}^{ 2} } \right) \, \left( {{\text{Z}}_{1 - \alpha /2} \pm {\text{ Z}}_{1 - \beta } } \right)/ \, \left( {{\text{X}}_{1}^{ - } \pm \,{\text{X}}_{2}^{ - } } \right)^{2}$$

$${\text{X1 }} = { 24}. 6,{\text{ X2 }} = { 22}. 6$$

$${\text{S1 }} = { 2}. 7,{\text{ S2 }} = { 2}. 4$$

$${\text{Z1}} - \alpha / 2 { } = { 1}. 9 6,{\text{ Z1}} - \beta \, = { 1}. 2 9$$

$${\text{n1 }} = {\text{ n2 }} = { 33}$$

The exclusion criteria were as follow: age under 18 years, a history of congenital anomalies in the cervical spine, a history of trauma to the neck with any severity, a history of structural, metabolic or rheumatologic disorders in the cervical spine, a history of any surgical or therapeutic intervention in the neck area.

**Fig. 1:**
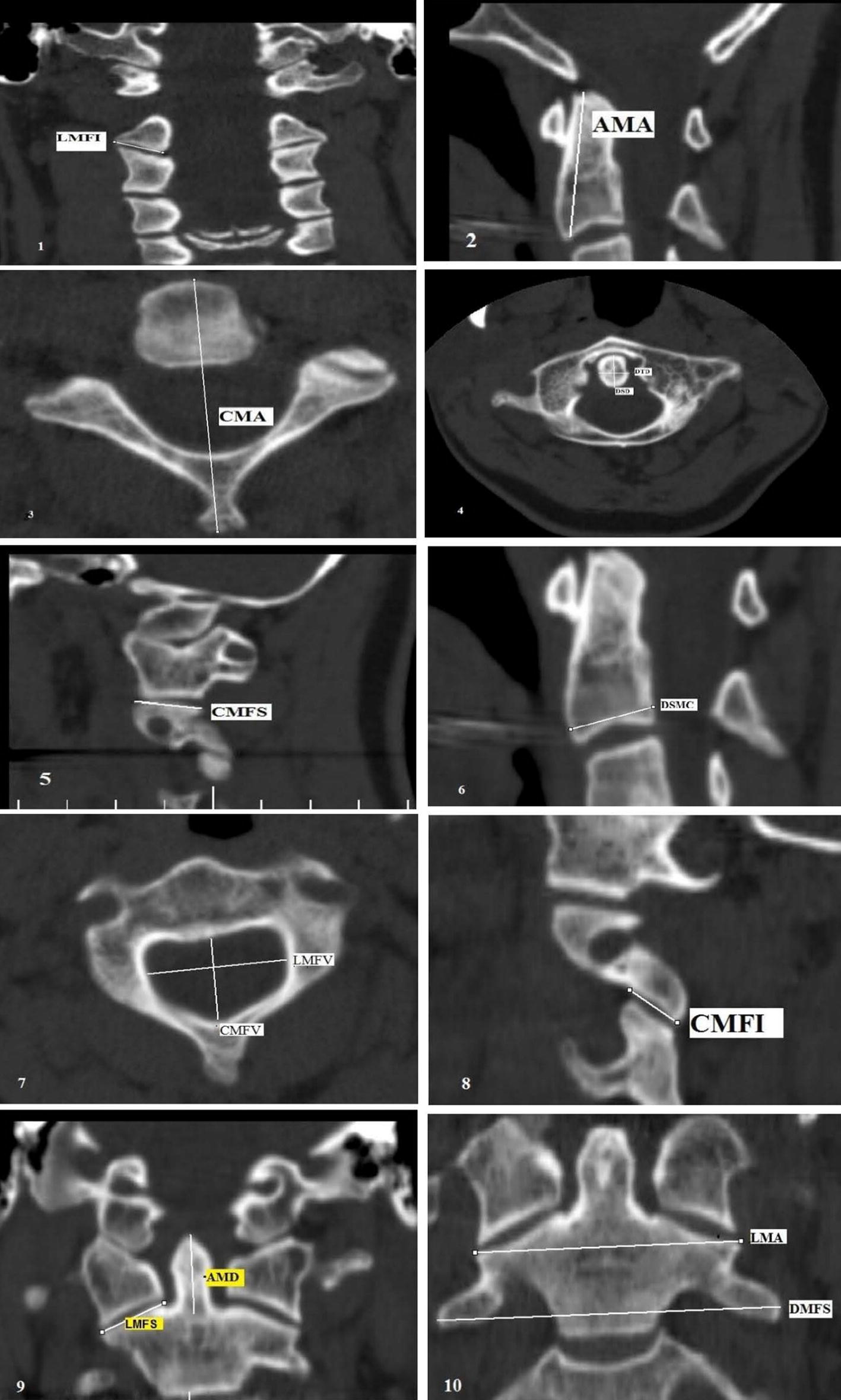
The Measured Dimensions of the Second Cervical Vertebrae in the (1) Coronal (LMFI): max width of the inf. Facet (2) AMA (max hight of the axis) (3) CMA. max length of the axis (4) Axial (DTD, DSD) odontoid process transverse diameter, odontoid process sagittal diameter 5) CMFS (max length of the sup. Facet) (6) DSMC. sagittal max body diameter (7) Axial (LMFV, CMFV) max width of the vertebral foramen, max length of the vertebral foramen (8) CMFI. (max length of the inf. Facet) (9) Cronal (AMD, LMFS) (max height of the odontoid process) (max width of the sup. Facet) (10) Coronal (LMA, DMFS): max width of the axis, max distance between the superior facets

The linear dimensions of the second cervical vertebrae were measured using three dimensional CT scan (Slice, Toshiba, Japan) with Multi-Planar Reconstruction (MPR) and Volume Rendering at sagittal and horizontal sections. The thickness of the slices was 1 mm. Fifteen indexes of the second cervical vertebrae were measured: Max height of the axis (AMA), Max length of the axis (CMA), Odontoid process sagittal diameter (DSD), Odontoid process transverse diameter (DTD), Max distance between the superior facets (DMFS), Max length of the sup. Facet (CMFS), Max width of sup. Facet (LMFS), Length of the vertebral foramen (CMFV), Sagittal max body diameter (DSMC), Max width of the vertebral foramen (LMFV), Max height of the odontoid process (AMD), Max transverse diameter of the body (DTMC), Max width of the axis (LMA), Max length of the inf. Facet (CMFI), and Max width of the inf. Facet (LMFI). Then, the evaluated indexes were compared in different age groups and the diagnostic accuracy of each index for height estimation was evaluated. Figure 1 shows the schematic view of the dimensions measured in the second cervical vertebrae.

### Statistical analysis

Data were analyzed using SPSS version 23. For quantitative variables, *T* test was used to compare the data, and the Pearson correlation test was used to investigate the relationship between height and second cervical vertebrae indexes. Values were significant at P < 0.05.

The Kolmogorov–Smirnov test (K-S) test was used to determine the normal distribution of data.

### Results

The mean age of the participants was 40.91 ± 14.85 years old and the age range was between 18 to 83 years. The mean age of female was 40.54 ± 14.07 years and the mean age of male was 41.82 ± 16.38 years. There was no significant difference in age between male and female (P = 0.938).

In the present study 15, indexes of the second cervical vertebrae were measured. To investigate the relationship between height and second cervical vertebrae indexes, due to the normal distribution of data, the Pearson correlation test was used in the total population, and the results are summarized in Table 1. In the total population, among the indexes for the second vertebrae, only the AMA (r = 0.470, P = 0.0001), CMA (r = 0.320, P = 0.007), and DSMC (r = 0.281, P = 0.019) had a strong and positive correlation with height.

**Table 1:** Correlation between height and indices related to the second cervical vertebrae in the total population

Indexes	Correlation coefficient	P value
AMA	0.470	0.0001
CMA	0.320	0.007
LMA	0.085	0.483
DSD	0.064	0.598
DTD	0.031	0.801
DMFS	0.202	0.093
CMFS	0.170	0.161
LMFS	0.126	0.298
CMFV	0.095	0.434
LMFV	0.048	0.694
AMD	0.133	0.271
DSMC	0.281	0.019
DTMC	0.089	0.462
CMFI	0.056	0.644
LMFI	0.223	0.063

To investigate the relationship between age and the indexes of the second cervical vertebrae, due to the normal distribution of data, the Pearson correlation test was used in the total population and each sex, and the results are summarized in Table 2. In the total population, only DTD, DMFS, and DSMC indexes of the second cervical vertebra had a weak and positive correlation with age. There was no significant correlation between age and the second cervical vertebra indexes in male. A moderate significant positive correlation was observed in female between age and CMA, LMA, DTD, DMFS, and CMFS indexes.

**Table 2:** Correlation between age and indexes related to the second cervical vertebra

Indexes	Male		Female		Total	
	Correlation coefficient	P value	Correlation coefficient	P value	Correlation coefficient	P value
AMA	− 0.046	0.792	0.125	0.474	0.014	0.91
CMA	− 0.115	0.511	0.383	0.023	0.135	0.267
LMA	0.087	0.618	0.361	0.033	0.242	0.043
DSD	0.132	0.449	0.119	0.494	0.107	0.38
DTD	0.030	0.864	0.353	0.037	0.240	0.046
DMFS	− 0.025	0.888	0.406	0.016	0.237	0.048
CMFS	0.001	0.997	0.388	0.021	0.209	0.083
LMFS	0.183	0.292	0.202	0.244	0.167	0.166
CMFV	− 0.198	0.254	0.069	0.696	− 0.067	0.582
LMFV	− 0.091	0.604	0.159	0.361	0.033	0.788
AMD	0.222	0.200	0.092	0.599	0.150	0.217
DSMC	0.318	0.063	0.321	0.060	0.272	0.023
DTMC	0.018	0.919	0.014	0.936	0.013	0.914
CMFI	0.168	0.335	0.244	0.158	0.211	0.80
LMFI	0.083	0.635	0.203	0.243	0.130	0.285

### Discussion

In this study, for the first time, the dimensions of the second cervical vertebrae were used in the determination of the height in the mature and living population of Iran using their cervical CT scan. The relationship between heights of persons with spinal vertebra morphology is one of the effective factors in identifying a person in forensic medicine. For this reason, in cases where only part of the body spine is available, the morphology of the vertebral column can explain the various skeletal features of a person, including his height.

In this study, 15 indexes of second cervical vertebra and their relationship with height were investigated. In 3 indexes, this difference was statistically significant. These three indexes were AMA, CMA, and DSMC. Torimitsu et al. evaluated the height estimation in Japanese by measuring second cervical vertebra using a multidetector computed tomography (MDCT). They showed that All measurements of the C2 including the length from the top of the dens to the anteroinferior point of the vertebral body (DA), the length from the top of the dens to the posterior point of the spinous process (DS), and the length from the anteroinferior point of the vertebral body to the posterior point of the spinous process (AS), were positively correlated with height. The highest correlation was detected for the DA (r = 0.762), and the lowest correlation was seen for AS (r = 0.684). Besides, the standard errors of the estimate were large. They showed the size of the C2 as measured with MDCT images may be useful for stature estimation [11]. In a study by Rodríguez et al. the height estimation was performed from the first and second cervical vertebrae in a Spanish population. The best results were gained in all three groups, males, females and total population, by the four measures of the first (Height of vertebra (V) + Interforaminal length (I)) and second (Greatest-diameter dens (DO) + Height of the odontoid (O)) cervical vertebrae. For estimating height for females the determination coefficient was somewhat higher by the measurements of C2 than C1. Though, for male both determination coefficients are equal [16].

In a study by Nagesh et al., the stature in South Indians was estimated from vertebral column length. According to the result of the logistic regression test in this study, the value of the correlation coefficient for height estimation from the cervical segment was 0.583 in males and 0.325 in females. This study showed that height estimates can be made from different parts of the spine, especially cervical vertebrae [10]. In a study by Wu RQ et al. in 2017, on 933 people in the population of the Yangtze River Delta, the dimensions and indices of the cervical vertebrae were evaluated and compared to find its relationship with the height. The analysis of the data showed that the size and dimensions of the neck bones were an appropriate method for the rapid, simple and accurate assessment of height for forensic experts [17]. In a study by Zhang et al. the anterior and posterior heights of C3-C7 were measured in the lateral films of the cervical vertebrae in the male population of Sichuan Han in China. For stature estimation between the cervical vertebrae and height, linear regression analysis was done to establish the regression equations. They showed that all equations were useful to estimate the body height of the adult males [18].

Preceding studies have revealed the accuracy of stature estimation using long bones [3, 5, 6], but most of the times in disaster, burn, and skeletal traumas it is not possible to collect the intact long bones and other more frequent, smaller and stronger bones like vertebrae should be considered.

Among the second cervical vertebrae indexes, 3 showed a correlation with height. These three indices were AMA, CMA, and DSMC. The results of the current study revealed the accuracy of linear dimensions of cervical vertebrae in determining the height of skeletal remains in the Iranian adult population.

## Limitation

Differences in the body’s dimensions can be seen as a confounding factor concerning the dimensions of the vertebrae with height. Because of the limitations in this study, it was not possible to study the body dimensions of the subjects, but in future studies, fitting the body to remove its confounding effect and finding independent variables that determine the height of the dimensions of the vertebrae are suggested.

## Data Availability

Data are available from corresponding author upon request.

## Abbreviations

MPR: Multi-Planar Reconstruction
AMA: max height of the axis
CMA: max length of the axis
DSD: odontoid process sagittal diameter
DTD: odontoid process transverse diameter
DMFS: max distance between the superior facets,
CMFS: max length of the sup. Facet
LMFS: max width of sup. Facet
CMFV: length of the vertebral foramen
DSMC: sagittal max body diameter
LMFV: max width of the vertebral foramen
AMD: max height of the odontoid process
DTMC: max transverse diameter of the body
LMA: max width of the axis
CMFI: max length of the inf. Facet
LMFI: max width of the inf. Facet
